# Factors for Visual Acuity Improvement After Anti-VEGF Treatment of Wet Age-Related Macular Degeneration in China: 12 Months Follow up

**DOI:** 10.3389/fmed.2021.735318

**Published:** 2021-11-11

**Authors:** Yan Lu, Wenzhi Huang, Yuehong Zhang, Xiongfei Huang, Xu Zhang, Haizhi Ma, Guoliang Ren, Feng Shi, Lihui Kuang, Shigang Yan, Shuke Luo, Junyan Zhang, Jingfang He, Weizhong Yang, Zongyin Gao, Yunxia Leng

**Affiliations:** ^1^Department of Ophthalmology, Foshan Second People's Hospital, Foshan, China; ^2^Department of Ophthalmology, Guangzhou First People's Hospital, South China University of Technology, Guangzhou, China; ^3^Bothwin Clinical Study Consultant, Redmond, WA, United States; ^4^Bothwin Clinical Study Consultant, Shanghai, China

**Keywords:** age-related macular degeneration, ranibizumab, conbercept, polypoidal choroidal vasculopathy (PCV), choroidal neovascularization (CNV)

## Abstract

**Purpose:** To evaluate the treatment solutions and effectiveness of intravitreal ranibizumab (RBZ) or conbercept in patients with wet age-related macular degeneration (wAMD) in a real-life setting in China.

**Methods:** The medical records of 368 patients with wAMD who started RBZ or conbercept treatment between 1 May 2014 and 30 April 2018 were evaluated. All patients were defined on fundus angiography at baseline to determine the subtype of AMD (PCV or CNV). We report visual acuity (VA) and central retinal thickness (CRT) measurements at baseline and 12 months.

**Results:** The average number of anti-VEGF injections was 2.1 ± 1.2. The BCVA improvement of these two groups was similar with a difference of 1.00 letter (95% CI: −1.4~3.4, *p* = 0.8505). At the end of the study, a BCVA increase of at least 5 letters was determined to be a satisfactory efficacy endpoint. Several factors were related to the possible improvement in the satisfactory efficacy endpoint, including female sex (OR 2.07, 95% CI 1.22~3.51), number of injections (OR 1.40, 95% CI 1.12~1.75) and VA change at the first month (OR 13.75, 95% CI 7.41~25.51). Additionally, some factors were related to the possible reduction in the satisfactory efficacy endpoint, including diabetes (OR 0.27, 95% CI 0.10~0.73) and disease history (OR 0.75, 95% CI 0.57~0.98).

**Conclusion:** Our study demonstrates that anti-VEGF drugs can effectively improve BCVA and reduce CRT in AMD patients. Sex, number of injections, VA change at the first month, diabetes and disease history are the most important factors affecting visual acuity.

## Introduction

Age-related macular degeneration (AMD) is a chronic degenerative disease that occurs in the macular area of the retina. The number of moderate-to-severe visual impairments caused by AMD is more than 8 million globally, and it is one of the main causes of irreversible visual impairment in people over 55 years old in developed countries (1, 2). The prevalence of AMD is increasing in China due to the accelerated aging of the population (3–5). The early stages of AMD are hidden, and progression is slow. If the lesion progresses to the macular area, the patient's vision will be severely reduced. This will acutely affect the patient's quality of life (6). The main pathological change in exudative AMD is the formation of choroidal neovascularization (CNV) in the macular region (1, 3, 7). Many studies have shown that overexpression of vascular endothelial growth factor (VEGF) plays a decisive role in its pathogenesis (8–11).

The last decade has seen the introduction of intravitreal anti-VEGF agents, which have revolutionized the treatment of wAMD, offering patients previously unachievable improvements in vision. Bevacizumab (avasin) was approved for use in the treatment of AMD in 2005 (12, 13), RBZ (lucentis) was approved in 2006 (14, 15), and conbercept was approved in 2013 (16, 17). RBZ (Lucentis) is a humanized monoclonal antibody fragment (18, 19). Conbercept, also known as KH902 (Chengdu Kanghong Biotech Co., Ltd, Sichuan, China), is a recombinant fusion protein designed as a decoy receptor and is composed of the VEGF-binding domains of VEGFR-1 and VEGFR-2 with the Fc portion of human IgG1 (16, 17).

Recently, a large number of real-world studies in developed countries in Europe and the Americas have shown that anti-VEGF requires regular and continuous administration for AMD to achieve better vision improvement (20). AMD treatment guidelines in many countries recommend 3 injections plus the Pro Re Nata (3+PRN) regimen to achieve better results.

However, the 3+PRN treatment regimen deviates significantly from real-world clinical use data, and the economic status of patients in China largely determines the number of injections. The deviation of treatment plans also led to a large difference in the efficacy of anti-VEGF treatments for AMD reported in China and other countries (21, 22).

Therefore, we designed this retrospective study to assess the usage and efficacy of intravitreal anti-VEGF (including RBZ and conbercept) in patients with untreated wAMD in real-world practice conditions in China.

## Methods

### Ethical Approval

The medical ethics committee of Second Affiliated Hospital of South China University of Technology and Foshan Second People's Hospital approved this study. The accession number was K-2018-123-02.

### Research Subjects

Patients diagnosed with wAMD who were treated in the Department of Ophthalmology at Second Affiliated Hospital of South China University of Technology or Foshan Second People's Hospital from 1 May 2014 to 30 April 2018 were recruited. These patients were diagnosed with wAMD by ophthalmologists according to fundus fluorescein angiography (FFA), indocyanine green angiography (ICGA) and optical coherence tomography (OCT) examination, including polypoidal choroidal vasculopathy (PCV) and CNV.

Study Design: All patients were continuously followed up at the Second Affiliated Hospital of South China University of Technology and at Foshan Second People's Hospital. They were treated at least once with intravitreal injections of anti-VEGF (RBZ or conbercept). During the follow-up process, it is recommended that patients visit the doctor every 4–8 weeks for at least 1 year. Patients were required to undergo best-corrected visual acuity (BCVA) assessment, slit-lamp examination, indirect ophthalmoscopy, fundus photography, and optical coherence tomography (OCT) each time. The standards and recommendations for retreatment also depend on the results of these examinations.

### The Inclusion Criteria

- Diagnosis of wAMD with completed follow up data.
- Age above 18 years.
- At least one treatment with anti-VEGF intraocular injection.
- Follow-up for at least 1 year after the first injection.

### The Exclusion Criteria

- Major ocular surgical procedures 3 months before the first injection.
- Myopia >-6.00 D.
- Ocular axial length >26 mm.
- Concurrent retinal vascular disorders in the studied eyes.
- All patients who underwent ocular surgery performed during the follow-up period, including cataract, glaucoma, vitreoretinal, macular laser grid photocoagulation, or YAG (neodymium-doped yttrium aluminum garnet) capsulotomy.

### Examinations

For all patients, the examinations included the BCVA based on the Snellen chart. The Snellen fraction was converted into an approximate Early Treatment Diabetic Retinopathy Study (ETDRS) equivalent letter score (23). Before anti-VEGF intravitreal injection, all patients had to be diagnosed with CNV or PCV by the results of FFA or ICGA (Heidelberg, Germany). A Cirrus HD-OCT 5000 (Zeiss, Germany) was used for OCT. The central retinal thickness (CRT) was measured before the first anti-VEGF injection and after the initial injection at 1, 2, 3, 6, and 12 months.

### Treatment Regimens

Before the initial anti-VEGF injection, patients were advised to take the continuous regimen of 3+PRN. Additional reinjections were given if any of the following changes were observed by the evaluating physician: (1) visual acuity loss of at least five letters and (2) an increase in OCT central retinal thickness of at least 100 μm. However, additional injections and treatment regimens were dependent on many factors, such as the patient's visual acuity improvement and economic status.

### Data Collection

The following data were collected retrospectively: the number of injections, intraocular pressure, BCVA, CRT, subtype of AMD, lesion diameter, duration, hypertension, diabetes, and demographic data. Each patient had baseline characteristics and data from the 12 months of follow-up. BCVA was recorded using the Snellen visual acuity chart, and the Snellen readings were converted into ETDRS letters for statistical analysis, according to Amoaku et al. (24).

### Statistical Analysis

Stata SE 13 (serial number 401306302851) was used for the statistical analysis. Continuous variables with normal distributions are presented as means and standard deviations. Continuous variables with abnormal distributions are continuous variables which were, respectively, presented as medians and interquartile ranges (IQRs). Baseline normal and abnormal quantitative data were respectively analyzed using student *T*-test and Wilcoxon rank-sum test. Categorical variables at baseline were analyzed using the chi-square test. For the outcomes analysis, multivariate logistic regression analysis was performed to identify the associations between dependent and independent variables. The results are expressed as adjusted odds ratios (ORs) with 95% confidence intervals (95% CIs). Significance was accepted as two-sided test with an alpha level of 0.05. Restricted cubic spline(RCS) analyses were performed by using EmpowerStats software (www.empowerstats.com, X&Y solutions, Inc. Boston MA).

Two-sided 95% confidence intervals was used to calculate sample size together with 95% power. Assuming 40% of patients with diabetics can improve their BCVA to 5 letters while OR was 4 for those without diabetics based on our clinical experiences. Since there were more patients without diabetes than those with, we used a 10:1 ratio to put their data into this study.

## Results

### Patient Characteristics

According to our inclusion criteria, 368 untreated wAMD (PCV/CNV) patients (368 eyes) were included. Among them, 223 were males, and 145 were females. All patients received intravitreal injections of RBZ or Conbercept at least once from May 2014 to November 2018. Each of these patients was followed up for at least 12 months. The mean age was 68.9 ± 9.5 years. A total of 781 anti-VEGF injections were used (605 RBZ and 176 conbercept). Table 1 shows the baseline characteristics and clinical data of all patients. The average number of intraocular anti-VEGF injections was 2.1 ± 1.2 (showed in Table 2).

**Table 1:** Baseline characteristics of all patients.

	**ALL**	**CNV**	**PCV**	***P***	**Conbercept**	**RBZ**	***P***
Patients (eyes)	368	233	135		88	280	
**Age (years)**							
*mean ± SD*	68.9 ± 9.5	69.5 ± 9.9	67.8 ± 8.9	0.098^*^	68.6 ± 8.9	69.0 ± 9.7	0.725^*^
*median (IQR)*	69 (61.5~76)	69 (62~77)	67 (61~75)		69 (62~75.5)	69 (61~76.5)	
*min~max*	40~95	40~95	48~90		50~90	40~95	
Female sex *n* (%)	145 (39.4%)	93 (39.9%)	52 (38.5%)	0.792^#^	31 (35.2%)	114 (40.7%)	0.358^#^
CNV/PCV	233/135	–	–		46/42	187/93	0.014^#^
Diabetes *n* (%)	33 (9.0%)	25 (10.7%)	8 (5.9%)	0.120^#^	8 (9.1%)	25 (8.9%)	0.963^#^
Hypertension *n* (%)	119 (32.3%)	74 (31.8%)	45 (33.3%)	0.756^#^	26 (30.0%)	93 (33.2%)	0.521^#^
Smoking *n* (%)	11 (3.0%)	4(1.7%)	7(5.2%)	0.060^#^	2(2.3%)	9(3.2%)	0.651^#^
**Disease history (month)**							
*mean ± SD*	10.2 ± 18.5	9.8 ± 17.9	11.0 ± 19.6	0.849^∧^	10.2 ± 19.7	10.2 ± 18.2	0.702^∧^
*median (IQR)*	3 (1~12)	3 (1~11)	3 (1~12)		3 (1~11.5)	3 (1~12)	
*min~max*	0.1~123	0.1~123	0.1~120		0.1~120	0.1~123	
**Diameter of lesion (μm)**							
*mean ± SD*	2495.5 ± 1559.5	2389.9 ± 1529.9	2677.8 ± 1598.6	0.067^∧^	2378.3 ± 1447.2	2532.3 ± 1593.8	0.452^∧^
*median (IQR)*	2,204 (1429.5~3266.5)	2,109 (1,428~3,043)	2,423 (1,434~3,712)		2,067 (1,417~3120.5)	2,264 (1434.5~3,338)	
*min~max*	92~12,700	107~12,700	92~7,597		92~6,549	107~12,700	
**BCVA (ETDRS letters)**							
*mean ± SD*	31.3 ± 13.3	31.6 ± 13.7	30.8 ± 12.7	0.905^∧^	31.6 ± 13.4	31.2 ± 13.3	0.906^∧^
*median (IQR)*	25 (23~40)	25 (22~40)	25 (23~40)		25 (22.5~40)	25 (23~40)	
*min~max*	20~75	20~75	20~75		20~70	20~75	
**CRT (mm)**							
*mean ± SD*	440.6 ± 228.6	420.7 ± 214.4	475.1 ± 248.4	0.008^∧^	448.6 ± 236.5	438.1 ± 226.5	0.869^∧^
*median (IQR)*	372 (277~541)	353 (269~522)	416 (321~590)		372 (275~566)	372.5 (285.5~535)	
*min~max*	100~1,632	100~1,221	123~1,632		143~1,238	100~1,632	
PDT *n* (%)	46 (12.5%)	13 (5.6%)	33 (24.4%)	<0.0001^#^	5 (5.7%)	41 (14.6%)	0.027^#^

* *Student t-test;*

∧ *Mann-Whitney U-test;*

# *chi-square*.

**Table 2:** Number of injections of all patients.

	**ALL**	**CNV**	**PCV**	**P**	**Conbercept**	**RBZ**	***P***
**Number of injection**							
*mean ± SD*	2.1 ± 1.2	2.2 ± 1.2	2.0 ± 1.2	0.052^∧^	1.8 ± 1.2	2.2 ± 1.2	0.001^∧^
*median (IQR)*	2 (1~3)	2 (1~3)	2 (1~3)		1 (1~2)	2 (1~3)	
*min~max*	1~8	1~8	1~8		1~7	1~8	
anti-VEGF injections Conbercept/RBZ	176/605	95/419	64/203	0.071^#^	–	–	–
Patient number in treatment group Conbercept/RBZ	88/280	46/187	42/93	0.014^#^	–	–	–
PDT *n* (%)	46 (12.5%)	13 (5.6%)	33 (24.4%)	<0.0001^#^	5 (5.7%)	41 (14.6%)	0.027^#^

∧ *Mann-Whitney U-test;*

# *chi-square*.

### BCVA Changes Compared With Baseline Values (ETDRS Letters)

**Figure 1:**
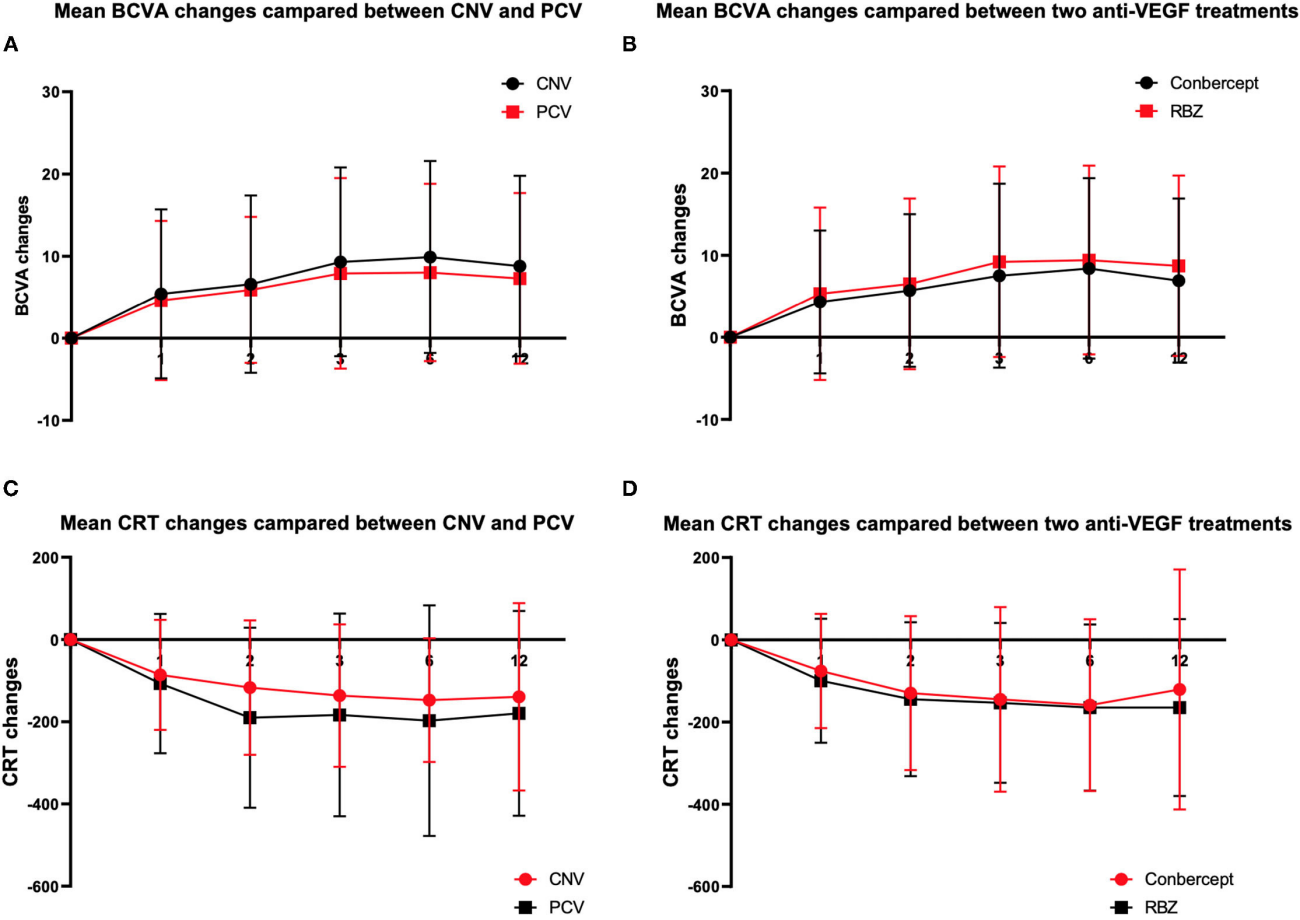
BCVA **(A,B)** and CRT **(C,D)** changes from baseline to month 12 in different grouping methods.

In this study, BCVA was 31.3 ± 13.3 letters at baseline with a median of 25. Table 2 shows that the mean changes in the BCVA letters at the 1st, 2nd, 3rd, 6th, and 12th months after the initial anti-VEGF injection were 5.09 ± 10.09, 6.33 ± 10.16, 8.77 ± 11.5, 9.18 ± 11.41, and 8.23 ± 10.80, respectively (Table 3, Figures 1A,B).

**Table 3:** Mean BCVA changes compared with baseline (ETDRS letters).

	**ALL**	**CNV**	**PCV**	***P***	**Conbercept**	**RBZ**	***P***
**Baseline**							
*mean ± SD*	31.3 ± 13.3	31.6 ± 13.7	30.8 ± 12.7	0.905^∧^	31.6 ± 13.4	31.2 ± 13.3	0.906^∧^
*median (IQR)*	25 (23~40)	25 (22~40)	25 (23~40)		25 (22.5~40)	25 (23~40)	
*min~max*	20~75	20~75	20~75		20~70	20~75	
**M1 change**							
*mean ± SD*	5.1 ± 10.1	5.4 ± 10.3	4.6 ± 9.7	0.840^∧^	4.3 ± 8.7	5.3 ± 10.5	0.851^∧^
*median (IQR)*	1 (0~9.5)	1 (0~10)	2 (0~8)		1.5 (0–7.5)	1 (0–11)	
*min~max*	−35~51	−35~50	−27~51		−23~30	−35~51	
**M2 change**							
*mean ± SD*	6.3 ± 10.2	6.6 ± 10.8	5.9 ± 8.9	0.544^∧^	5.7 ± 9.3	6.5 ± 10.4	0.853^∧^
*median (IQR)*	3 (0–13)	3 (0–13)	2 (0–13)		3 (0–8.75)	3 (0–13)	
*min~max*	−35~53	−35~53	−17~37		−23~37	−35~53	
**M3 change**							
*mean ± SD*	8.7 ± 11.5	9.3 ± 11.5	7.9 ± 11.6	0.123^∧^	7.5 ± 11.2	9.2 ± 11.6	0.177^∧^
*median (IQR)*	5 (0–15)	7 (2–15)	4 (0–15)		4 (0–15)	6 (1–16)	
*min~max*	−27~52	−27~52	−20~52		−23~52	−27~52	
**M6 change**							
*mean ± SD*	9.2 ± 11.4	9.9 ± 11.7	8.0 ± 10.8	0.113^∧^	8.4 ± 11.0	9.4 ± 11.5	0.537^∧^
*median (IQR)*	5.75 (1–15)	7 (2–15)	4 (0–15)		4 (0–15)	6 (1–15)	
*min~max*	−27~52	−27~52	−18~37		−23~33	−27~52	
**M12 change**							
*mean ± SD*	8.2 ± 10.8	8.8 ± 11.0	7.3 ± 10.4	0.131^∧^	6.9 ± 10.0	8.7 ± 11.0	0.156^∧^
*median (IQR)*	5 (0–15)	5 (1–15)	4 (0–15)		4 (0–14)	5 (0–15)	
*min~max*	−27~50	−27~50	−19~37		−23~32	−27~50	

∧ *Mann-Whitney U-test*.

### CRT Changes Compare With Baseline Values (μm)

CRT was 440.6 ± 228.6 μm at baseline with a median of 372. Table 2 also shows that the mean changes in CRT at the 1st, 2nd, 3rd, 6th, and 12th months after the initial anti-VEGF injection were −93.9 ± 148.1, −142.8 ± 187.1, −151.8 ± 200.9, −163.6 ± 203.0, and −154.18 ± 236.3, respectively (Table 4, Figures 1C,D).

**Table 4:** Mean CRT changes compared with baseline (μm).

	**ALL**	**CNV**	**PCV**	***P***	**Conbercept**	**RBZ**	***P***
**Baseline**							
*mean ± SD*	440.6 ± 228.6	420.7 ± 214.4	475.1 ± 248.4	0.008^∧^	448.6 ± 236.5	438.1 ± 226.5	0.869^∧^
*median (IQR)*	372 (277~541)	353 (269~522)	416 (321~590)		372 (275~566)	372.5 (285.5~535)	
*min~max*	100~1,632	100~1,221	123~1,632		143~1,238	100~1,632	
**M1 change**							
*mean ± SD*	−93.9 ± 148.1	−86.2 ± 134.0	−107.3 ± 169.5	0.071^∧^	−75.8 ± 138.9	−99.7 ± 150.7	0.316^∧^
*median (IQR)*	−61 (−143.5~-16)	−53 (−116~-16)	−79 (−161~-16)		−56.5 (−117~-4)	−61 (−147~-19.5)	
*min~max*	−835~365	−835~262	−800~365		−562~344	−835~365	
**M2 change**							
*mean ± SD*	−141.8 ± 187.1	−116.9 ± 163.5	−190.2 ± 219.11	0.002^∧^	−129.8 ± 187.2	−144.4 ± 187.5	0.535^∧^
*median (IQR)*	−82 (−210~-27)	−64 (−176~-21)	−125 (−259~-53)		−81 (−154~-6)	−82 (−211~-27)	
*min~max*	−977~151	−962~151	−977~111		−893~90	−977~151	
**M3 change**							
*mean ± SD*	−151.8 ± 200.9	−136.5 ± 173.4	−183.4 ± 246.5	0.142^∧^	−145.1 ± 224.8	−153.6 ± 194.6	0.238^∧^
*median (IQR)*	−89 (−218~-45)	−81 (−191~-44)	−126 (−266~-51)		−70.5 (−232~-4)	−96 (−209~-51)	
*min~max*	−1,344~387	−807~387	−1,344~191		−908~134	−1,344~387	
**M6 change**							
*mean ± SD*	−163.6 ± 203.1	−147.3 ± 150.7	−197.4 ± 280.7	0.162^∧^	−158.8 ± 208.7	−164.7 ± 202.3	0.463^∧^
*median (IQR)*	−106.5 (−215~-51)	−100 (−203~-47)	−140 (−243~-55)		−95 (−197~-21)	−111 (−224~-54)	
*min~max*	−1,392~385	−700~108	−1,392~385		−717~212	−1,392~385	
**M12 change**							
*mean ± SD*	−154.2 ± 236.3	−139.4 ± 227.8	−179.7 ± 249.2	0.078^∧^	−120.8 ± 292.0	−164.7 ± 215.4	0.218^∧^
*median (IQR)*	−110.5 (−228.5~-48.5)	−96 (−213~-48)	−138 (−238~-49)		−102 (−199.5~-29)	−116 (−238.5~-49)	
*min~max*	−1,392–2,013	−988~2,013	−1,392~398		−908~2,013	−1,392~512	

∧ *Mann-Whitney U-test*.

### Comparative Analysis of the RBZ Group With Conbercept Group

Two anti-VEGF drugs, RBZ and conbercept, were used in this analysis. Two hundred eighty patients were treated with RBZ, while the other 88 patients were treated with conbercept. There were no differences between these two groups at baseline in terms of epidemiological data and disease characteristics (Table 1). However, the average injection numbers of the two drugs were different (Table 2). The average number of injections for RBZ was 2.2 ± 1.2 and that for Conbercept was 1.8 ± 1.2 (*p* = 0.001). The difference between the two groups was 0.41 (SE 0.15, 95% CI 0.12~0.70).

Analysis of the BCVA improvement with the two treatment regimens (Table 3) showed that in the first month after the initial injection, the RBZ group increased by 5.3 ± 10.5 (median 1) letters, while the conbercept group increased by 4.3 ± 8.7 (median 1.5) letters. The BCVA improvement of these two groups was similar with a difference of 1.00 letter (95% CI: −1.4~3.4, *p* = 0.8505). At the 12th month after the initial injection, the RBZ group increased by 8.7 ± 11.0 (median 5) letters, and the conbercept group increased by 6.9 ± 10.0 (median 4) letters. The BCVA improvement of these two groups was similar with a difference of 1.8 (95% CI −0.8 ~4.4, *p* = 0.1561). A comparative analysis of CRT changes showed that during the first month after the initial injection, the CRT change in the RBZ group was −99.7 ± 150.7 (median −61) mm and that in the conbercept group was −75.8 ± 138.9 (median −56.5) mm. The CRT change value of the RBZ group at the 12th month after the initial injection was −164.7 ± 215.4 (median −116) mm, while it was −120.8 ± 292.0 (median −102) mm in the conbercept group. There was no significant difference between the two groups; the difference was 43.9 μm (95% CI: −12.7~100.6).

### Comparative Analysis of PCV and CNV

This study included two subtypes of wAMD. One hundred thirty-five (36.68%) of all patients were diagnosed with PCV, and the other 233 (63.32%) were diagnosed with CNV. There was no significant difference in epidemiological data between the two groups. The average injection number of patients with PCV was 2.0 ± 1.2, and the average injection number for those with CNV was 2.2 ± 1.2, as shown in Table 2.

A comparative analysis of the BCVA improvement of the two subtype groups at the 1st month after the initial injection showed that the BCVA increased by 4.6 ± 9.7 (median 2) letters among PCV patients and 5.4 ± 10.3 (median 1) letters among CNV patients. The BCVA increased by 7.30 ± 10.41 (median 4) letters at the 12th month for these PCV patients and 8.8 ± 11.0 (median 5) letters for the CNV patients (Table 3).

There was no significant difference between the two groups. At the 1st month after the initial injection, CRT changes were −107.3 ± 169.5 (median −79) μm for PCV patients and −86.2 ± 134.0 (median −53) μm for CNV patients. At the 12th month, CRT changes were −179.7 ± 249.2 (−138) μm for PCV patients and −139.4 ± 227.8 (median −96) μm for CNV patients. There was no significant difference between the two groups (Table 3).

### Univariate and Multivariate Analyses

BCVA improvement of more than 5 letters on the ETDRS chart was considered to be a satisfactory efficacy endpoint. The univariate and multivariate analyses are shown below in Table 5.

**Table 5:** Univariate and multivariate analyses.

	**Univariate**			**Multivariate**		
	**OR**	**95% CI**	***p***	**OR**	**95% CI**	***p***
Sex, F vs. M	1.44	0.95~2.20	0.087	2.07	1.22~3.51	0.007
Age (above 65 years)	0.76	0.50~1.17	0.213	0.80	0.47~1.36	0.402
Smoking	1.49	0.45~4.99	0.514	1.94	0.44~8.45	0.379
Hypertension	1.26	0.81~1.96	0.298	1.53	0.86~2.72	0.150
Diabetics	0.50	0.23~1.09	0.083	0.27	0.10~0.73	0.010
PCV/CNV	0.77	0.50~1.18	0.230	0.82	0.47~1.44	0.495
AMD disease history (more than 12 months; 3–12 months; 1–3 months; 1 and below)	0.77	0.64~0.94	0.011	0.75	0.57~0.98	0.033
PDT	1.04	0.56~1.93	0.905	0.79	0.35~1.79	0.577
Anti-VEGF-RBZ/Conbercept	1.69	1.03~2.77	0.039	1.48	0.79~2.73	0.222
Number of injections	1.13	0.96~1.34	0.150	1.40	1.12~1.75	0.003
Diameter of lesion above 500	0.14	0.03~0.64	0.011	0.24	0.04~1.34	0.105
Baseline OCT	1.00	1.00~1.00	0.005	1.00	1.00~1.00	0.208
VA change at the 1st month above 5 letters.	10.92	6.33~18.86	<0.001	13.75	7.41~25.51	<0.001

*Adjusted by sex, age, smoking status, hypertension, diabetics, PCV/CNV, AMD disease history, PDT usage, kind of anti-VEGF, diameter of lesion, baseline OCT, and whether VA changing at the 1st month above 5 letters*.

Several factors were related to the increased probability of reaching this satisfactory efficacy endpoint, including female sex (OR 2.07, 95% CI 1.22~3.51), number of injections (OR 1.40, 95% CI 1.12~1.75) and VA change at the first month (OR 13.75, 95% CI 7.41~25.51). Additionally, some factors were related to reducing the probability of reaching a satisfactory efficacy endpoint, including diabetes (OR 0.27, 95% CI 0.10~0.73) and AMD disease history (OR 0.75, 95% CI 0.57~0.98).

**Figure 2:**
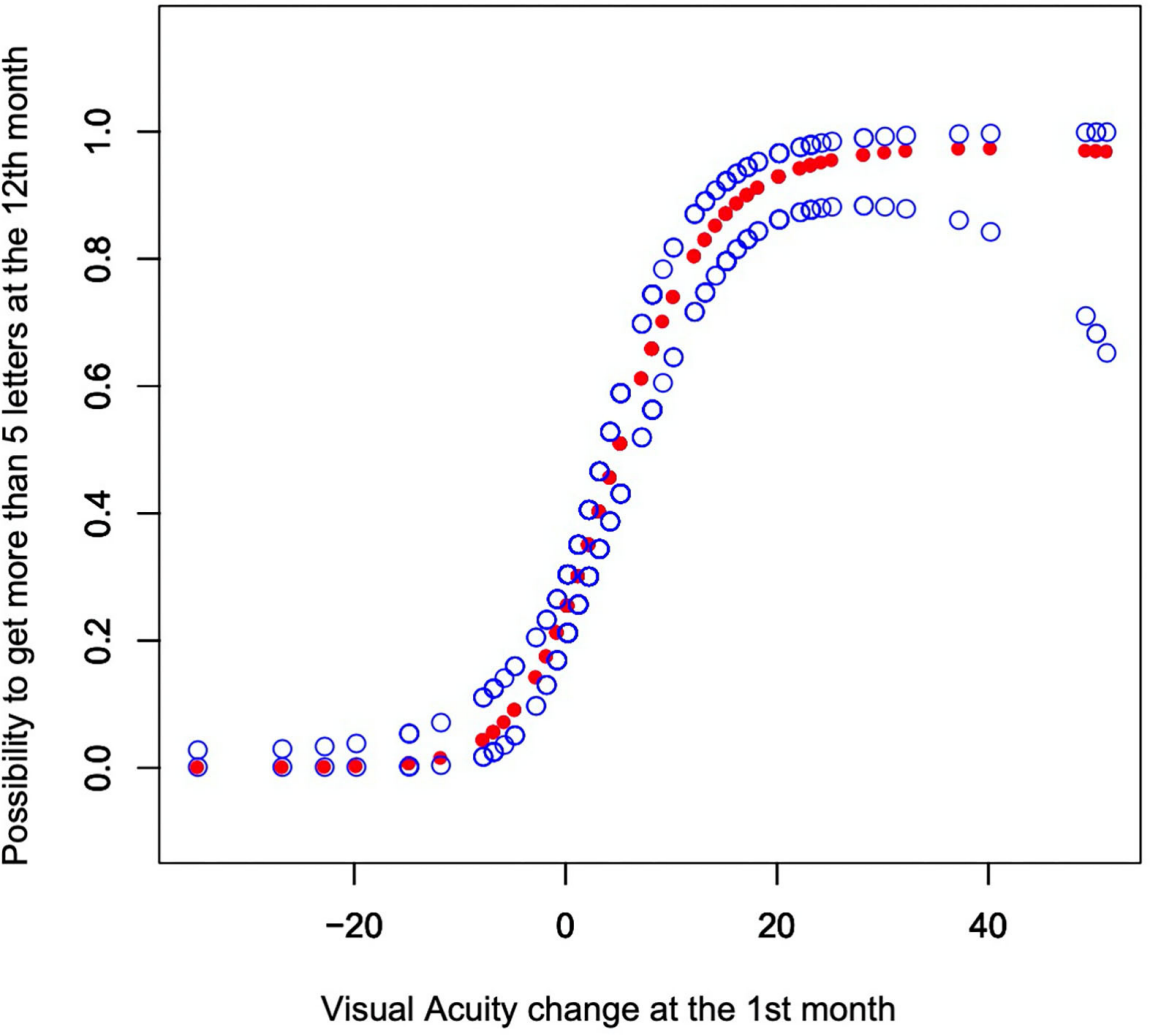
The y-axis shows the possibility to get more than 5 letters at the 12th month with the 95% CI. The model was adjusted by sex, age, smoking status, hypertension, diabetes, PCV or CNV, history of wet AMD, PDT (photodynamic therapy), treatment groups, number of injections, diameter of lesions, and baseline OCT.

RCS analyses generated a curve showing that those patients whose VA was not decreased at the 1st month had a higher probability of gaining more than 5 letters at month 12 (*p* < 0.001 Figure 2).

## Discussion

This was a real-life study of anti-VEGF therapy for wAMD between 2014 and 2018 in Guangdong Province, China, which reported changes in the BCVA and CRT of patients from initial baseline to the 12th month. Our results confirmed that BCVA and anatomical benefits could be maintained for at least 12 months after intravitreal injection of anti-VEGF. This outcome is consistent with the results of most previous anti-VEGF studies in the treatment of wAMD (25–28).

Many previous studies have shown more effectiveness of anti-VEGF in the treatment of wAMD if patients accepted the 3 + PRN or 5 + 3 + 2 in the 3-year treatment regimen (29–31). However, in China, these two anti-VEGF drugs were not approved by health insurance for use in patients with wAMD until May 2019. Prior to this date, the large cost of high-priced drugs greatly affected the patient's treatment compliance and compromised data integrity (32, 33). Therefore, the anti-VEGF treatment regimen and its efficacy in wAMD patients in China before 2019 were different from the data reported worldwide. For example, in our study, the mean number of intravitreal injections of anti-VEGF was only 2.1, and only 37.5% of patients completed the treatment regimen of 3+PRN, which was far lower than the mean number of injections reported in European countries (34–37).

The first-year result of the LUMINOUS study with 3,379 patients globally showed that the mean number of intravitreal injections was 5.0, and VA increased by 3.1 letters on average (38). In China (92 patients), the average number of intravitreal injections was 2.9, and VA increased by 1.1 letters on average. In Canada (376 patients), the average number of intravitreal injections was 7.0, with VA increasing by an average of 2.5 letters. In Germany (128 patients), the average number of intravitreal injections was 5.2, with VA increasing by an average of 2.3 letters. In South Korea (52 patients), the average number of intravitreal injections was 5.2, and VA increased by an average of 9.8 letters. The average number of injections and the increase in VA letters at 1 year in our study results were different from the multinational data in the LUMINOUS study.

Conbercept is an anti-VEGF drug produced in China that is permitted for the treatment of wAMD. However, the efficacy of conbercept in wAMD still lacks data support compared with other anti-VEGF drugs (39–42). In this study, we compared RBZ and conbercept, two kinds of anti-VEGF drugs. The results showed that the mean number of injections for RBZ was slightly higher than that for conbercept and that the BCVA improvement of RBZ was better than that of conbercept. However, there were no significant differences between the two groups. This indicates that RBZ and conbercept have similar efficacy in wAMD. The reason for the difference may be that more injections lead to better visual benefits. Our result was similar to the results of a randomized controlled study between RBZ and aflibercept. Many real-world studies did not show any significant difference in VA improvement between RBZ and aflibercept (43–47).

PCV and CNV are two subtypes of wAMD. They have many common clinical characteristics and risk factors, but there are also many different epidemiological characteristics, indicating that they have different pathophysiological processes (48–50). These differences have led to a certain difference in the therapeutic effect between intravitreal injection of anti-VEGF drugs on PCV and CNV (51, 52). In epidemiology, compared to CNV secondary to AMD in Western populations, polypoidal PCV appears to be the main subtype of exudative AMD in Asian populations (53).

Our study included 135 PCV and 233 CNV cases. Our analysis found that the average number of injections for PCV was lower than that for CNV. However, the changes in BCVA and CRT in CNV were better than those in PCV. This finding is consistent with previous reports in the relevant literature (54).

At the end of the treatment in the 12th month, BCVA improved more than 5 letters on the ETDRS chart and was considered satisfactory efficacy. Univariate and multivariate analyses showed that females were more likely to experience good treatment effects than males. Moreover, increasing the number of injections has better vision benefits. This result is consistent with a number of studies (33, 38, 55).

Because this was a retrospective study, there were some limitations. First, we found that treatment was more effective in women than men. This may be related to the fact that Chinese women are more likely to follow the doctor's advice on matters such as less alcohol consumption and less tobacco usage, but we did not collect data on the frequency or amount of alcohol consumed. Although our results showed that tobacco had no significant effect on the efficacy of wAMD, the interaction between alcohol and tobacco was not included. Additionally, conbercept has been used only since the beginning of 2016, causing the sample size for conbercept to be smaller than that for RBZ. Therefore, the efficacy comparison between RBZ and conbercept still needs to be confirmed by further studies, especially RCTs.

Overall, this study is about the actual application of anti-VEGF in the treatment of wAMD among Chinese patients from 2014 to 2018. The Chinese medical insurance system did not reimburse such wAMD patients due to the cost of anti-VEGF treatment during that period. The anti-VEGF treatment of AMD patients was largely limited by their financial status. Therefore, the average number of injections for AMD in this study was much lower than that in developed countries. However, since 2019, anti-VEGF treatment for AMD has entered the era of comprehensive medical insurance payment in China. AMD patients in China can be more active in the selection of anti-VEGF treatment, which may produce better treatment results.

## Data Availability Statement

The original contributions presented in the study are included in the article/Supplementary Material, further inquiries can be directed to the corresponding author/s.

## Ethics Statement

The studies involving human participants were reviewed and approved by the Medical Ethics Committee of the Second Affiliated Hospital of South China University of Technology and Foshan Second People's Hospital. Written informed consent to participate in this study was provided by the participants' legal guardian/next of kin.

## Author Contributions

YLe and YLu contributed to the design, performed the experiments, and discussed the results. JZ and JH analyzed the data and prepared the tables and figures for the manuscript. FS, HM, and GR performed the medical record data collection. WH, LK, WY, XH, SY, and ZG followed up with the patients. All authors reviewed the manuscript.

## Funding

This research was supported by the Natural Science Foundation of Guangdong Province (No. 2018A030313761).

## Conflict of Interest

The authors declare that the research was conducted in the absence of any commercial or financial relationships that could be construed as a potential conflict of interest.

## Publisher's Note

All claims expressed in this article are solely those of the authors and do not necessarily represent those of their affiliated organizations, or those of the publisher, the editors and the reviewers. Any product that may be evaluated in this article, or claim that may be made by its manufacturer, is not guaranteed or endorsed by the publisher.
