# Supplementary material for: Factors for Visual Acuity Improvement After Anti-VEGF Treatment of Wet Age-Related Macular Degeneration in China: 12 Months Follow up
Source: Front Med (Lausanne). 2021 Nov 11;8:735318. doi: 10.3389/fmed.2021.735318 (PMC8632047; doi:10.3389/fmed.2021.735318)
Supplement: Supplementary file 1 [file Data_Sheet_1.PDF]

编码<br>ID	性别<br>男=1 女=2<br>sex	年龄/y<br>age	吸煙<br>不吸煙=0 吸煙=1 (11 人)<br>smoke	HBP<br>無=0 有=1<br>HBP	DM<br>無=0 有=1 (33 人)<br>DM	医保/Y<br>N=0 Y=1<br>Insurance
1	1	73	0	0	0	Y
2	2	72	0	0	0	Y
3	1	95	0	0	0	Y
4	1	56	0	0	0	Y
5	1	58	0	0	0	Y
6	2	84	0	0	0	Y
7	2	84	0	0	0	N
8	1	67	0	0	0	Y
9	1	65	0	1	0	Y
10	1	82	0	1	0	Y
11	1	82	0	1	0	Y
12	2	67	0	1	0	Y
13	1	80	0	0	0	N
14	2	60	0	0	0	Y
15	2	70	0	0	0	Y
16	1	61	0	0	0	Y
17	1	65	0	0	0	Y
18	2	69	0	1	0	Y
19	1	61	0	1	0	Y
20	1	80	0	0	0	Y
21	2	84	0	0	0	N
22	1	62	0	1	0	Y
23	1	83	0	1	0	N
24	2	75	0	0	0	Y
25	2	74	0	0	0	Y
26	2	71	0	0	0	Y
27	2	75	0	1	0	N
28	2	75	0	1	0	Y
29	1	62	0	0	0	Y
30	1	77	0	0	0	Y
31	2	55	0	0	0	N
32	1	60	0	0	0	N
33	1	69	0	1	0	Y
34	2	65	0	1	0	Y
35	1	85	0	1	1	Y
36	1	61	0	0	0	Y
37	1	70	0	0	0	N
38	1	80	0	1	0	Y
39	2	81	0	0	0	Y
40	1	69	0	0	0	Y
41	1	76	0	0	0	Y
42	2	77	0	0	0	Y
43	1	69	0	0	0	Y
44	1	71	0	1	0	Y
45	1	86	0	0	0	Y
46	2	57	0	0	0	N
47	1	69	0	0	0	Y

48	2	82	0	0	1	Y
49	2	74	0	0	0	N
50	1	72	0	0	0	Y
51	1	65	0	0	0	Y
52	1	64	0	0	0	Y
53	1	70	0	1	0	Y
54	1	65	0	0	0	Y
55	1	68	0	1	0	Y
56	1	49	0	1	0	Y
57	2	74	0	0	0	Y
58	2	56	0	0	0	Y
59	1	85	0	1	0	Y
60	1	83	0	0	0	N
61	1	75	0	1	1	Y
62	2	64	0	1	0	Y
63	1	69	0	0	0	Y
64	1	78	0	1	0	Y
65	1	78	0	1	0	Y
66	2	51	0	0	0	Y
67	1	54	0	0	0	Y
68	1	80	0	0	0	Y
69	1	77	0	0	0	Y
70	1	75	0	0	0	Y
71	1	62	0	0	0	Y
72	1	81	0	0	0	Y
73	1	73	0	1	0	Y
74	2	73	0	0	0	Y
75	1	84	0	0	0	Y
76	1	73	0	1	0	Y
77	1	53	1	1	0	Y
78	1	54	0	0	0	Y
79	1	79	0	0	0	Y
80	1	77	0	0	0	Y
81	1	60	0	1	0	Y
82	1	53	0	0	0	Y
83	2	61	0	1	1	Y
84	1	80	0	0	0	Y
85	1	80	0	0	0	Y
86	2	65	0	0	0	Y
87	1	67	0	0	0	Y
88	2	71	0	0	0	N
89	1	66	0	1	0	Y
90	1	54	0	0	0	Y
91	1	71	0	1	0	Y
92	1	81	0	0	0	Y
93	1	74	1	0	0	Y
94	1	69	0	0	0	Y
95	1	70	0	0	1	N
96	1	80	0	0	0	Y
97	1	62	0	0	0	Y
98	1	67	0	1	0	Y

99	1	53	0	1	0	Y
100	1	68	0	0	0	Y
101	1	57	0	0	0	Y
102	1	72	0	0	0	Y
103	1	77	0	0	0	Y
104	1	76	0	0	0	Y
105	2	86	0	1	1	Y
106	1	69	0	1	1	Y
107	1	74	1	1	0	Y
108	2	70	0	0	0	Y
109	2	57	0	0	0	Y
110	2	65	0	0	0	Y
111	1	71	0	1	1	Y
112	2	58	0	0	0	Y
113	2	91	0	0	0	Y
114	1	64	0	0	0	Y
115	1	60	0	0	0	Y
116	2	88	0	1	0	Y
117	2	60	0	0	0	Y
118	1	75	0	0	0	Y
119	2	79	0	0	0	Y
120	1	56	0	1	0	Y
121	1	75	0	0	0	Y
122	2	54	0	0	0	Y
123	2	73	0	0	0	Y
124	1	57	0	0	0	Y
125	2	61	0	0	0	Y
126	1	80	0	0	0	N
127	1	61	0	0	0	Y
128	1	79	0	0	0	Y
129	1	85	0	1	0	Y
130	2	72	0	1	0	Y
131	2	69	0	1	0	Y
132	2	72	0	0	0	Y
133	1	76	0	0	0	Y
134	1	76	0	0	0	Y
135	1	68	0	0	0	Y
136	1	69	0	0	0	Y
137	2	70	0	0	0	Y
138	2	65	0	0	0	Y
139	1	58	0	0	0	N
140	1	67	0	0	0	Y
141	2	59	0	0	0	Y
142	1	53	0	0	0	Y
143	2	62	0	0	0	N
144	1	71	0	0	0	Y
145	2	61	0	0	0	N
146	2	53	0	0	0	Y
147	1	66	0	1	0	Y
148	2	64	0	0	0	Y
149	1	52	0	0	0	Y

150	1	59	0	0	0	Y
151	1	68	0	0	0	Y
152	1	67	0	1	0	Y
153	1	80	0	1	0	Y
154	1	64	0	0	0	Y
155	2	67	0	0	0	N
156	2	58	0	0	0	N
157	1	83	0	0	1	Y
158	1	73	0	0	0	Y
159	2	53	0	0	0	Y
160	2	77	0	0	0	Y
161	1	60	0	0	1	Y
162	2	57	0	1	0	Y
163	2	70	0	0	0	Y
164	2	77	0	1	0	Y
165	1	61	0	0	0	Y
166	1	61	0	0	0	Y
167	2	71	0	1	0	Y
168	2	74	0	1	1	Y
169	2	72	0	1	1	Y
170	1	64	0	0	0	Y
171	2	75	0	0	0	Y
172	2	68	0	0	0	Y
173	1	74	0	0	0	Y
174	1	59	0	0	0	Y
175	1	60	0	0	0	Y
176	1	76	0	0	0	Y
177	1	80	0	0	1	Y
178	1	82	0	0	0	Y
179	2	62	0	1	0	Y
180	1	73	0	0	0	N
181	1	65	0	1	0	Y
182	1	65	0	0	1	Y
183	2	72	0	1	0	Y
184	1	56	0	0	0	Y
185	2	64	0	1	0	Y
186	1	69	0	0	0	Y
187	1	82	0	1	1	Y
188	2	61	0	0	0	N
189	1	80	0	0	0	Y
190	2	76	0	1	0	Y
191	1	69	0	0	0	Y
192	1	71	0	1	1	Y
193	1	61	0	0	1	Y
194	1	65	0	0	0	N
195	2	52	0	0	0	Y
196	2	56	0	0	0	Y
197	2	77	0	0	0	Y
198	1	54	0	0	0	Y
199	2	73	0	0	0	Y
200	2	62	0	0	0	Y
201	2	60	0	0	0	Y

202	2	65	0	0	0	Y
203	2	63	0	0	0	N
204	1	52	0	0	0	Y
205	1	75	0	1	0	Y
206	1	62	0	0	0	Y
207	2	52	0	0	0	Y
208	2	82	0	0	0	Y
209	2	55	0	0	0	Y
210	2	69	0	0	0	Y
211	2	58	0	0	0	Y
212	1	61	0	1	0	N
213	2	61	0	0	0	Y
214	1	60	0	1	0	Y
215	2	76	0	0	0	Y
216	1	77	0	0	0	Y
217	1	71	0	0	0	N
218	2	68	0	1	0	Y
219	1	85	0	0	0	Y
220	1	54	0	0	0	Y
221	1	66	0	0	0	Y
222	1	52	0	1	0	Y
223	2	58	0	0	0	Y
224	2	61	0	0	0	Y
225	1	71	0	0	0	Y
226	1	74	0	1	0	Y
227	2	65	0	0	0	Y
228	2	74	0	0	0	Y
229	2	69	0	1	1	Y
230	1	77	0	0	0	Y
231	1	58	0	0	0	Y
232	1	74	0	0	0	Y
233	2	63	0	1	1	Y
234	1	65	0	0	0	Y
235	1	72	0	0	0	Y
236	2	68	0	0	0	Y
237	1	77	1	1	0	Y
238	1	75	0	0	0	Y
239	1	80	0	0	0	Y
240	2	78	0	0	0	Y
241	2	57	0	0	0	Y
242	1	65	0	1	1	Y
243	1	65	0	1	0	N
244	2	78	0	0	0	Y
245	1	80	0	0	0	Y
246	2	81	0	1	0	Y
247	2	50	0	0	0	Y
248	1	69	1	0	0	Y
249	1	67	0	0	0	N
250	2	60	0	0	0	N
251	2	56	0	1	1	Y

252	2	77	0	1	0	Y
253	1	84	0	1	0	Y
254	1	75	0	0	0	Y
255	2	61	0	1	0	Y
256	2	64	0	0	0	Y
257	1	71	0	0	0	N
258	1	55	0	1	1	Y
259	1	66	0	0	0	Y
260	1	75	0	0	0	Y
261	2	66	0	0	0	N
262	1	72	0	0	0	Y
263	1	55	0	0	0	Y
264	2	66	0	1	0	Y
265	1	78	1	1	0	Y
266	2	80	0	1	0	Y
267	1	51	0	0	0	Y
268	1	74	0	1	0	N
269	1	57	0	0	0	Y
270	2	62	0	0	0	N
271	2	60	0	1	0	Y
272	2	77	0	1	0	N
273	2	77	0	0	0	N
274	2	86	0	1	1	Y
275	2	65	0	0	0	Y
276	2	40	0	1	1	N
277	1	85	0	0	0	Y
278	1	74	0	1	0	Y
279	1	85	0	0	0	N
280	1	73	1	0	0	Y
281	1	81	0	0	0	Y
282	1	71	0	0	0	Y
283	2	63	0	0	0	Y
284	1	75	0	0	0	Y
285	2	64	0	0	0	Y
286	2	68	0	1	0	Y
287	1	70	0	1	0	Y
288	1	76	0	1	0	Y
289	2	65	0	0	0	Y
290	1	81	0	0	0	Y
291	2	55	0	0	0	N
292	1	53	0	0	0	Y
293	2	87	0	0	0	Y
294	1	76	0	0	0	Y
295	1	79	0	1	0	Y
296	1	72	1	1	0	Y
297	1	80	0	1	1	Y
298	1	59	0	0	0	N
299	2	61	0	1	0	Y
300	1	67	0	0	0	N
301	2	78	0	0	0	Y
302	1	61	0	1	0	Y
303	2	79	0	1	0	Y

304	1	62	0	0	0	Y
305	1	55	0	1	0	Y
306	1	83	0	0	0	Y
307	1	69	0	1	1	Y
308	1	82	0	0	0	Y
309	1	67	0	1	0	N
310	1	68	0	0	0	Y
311	2	76	0	0	0	Y
312	1	68	0	0	0	N
313	1	67	0	1	1	Y
314	1	81	0	0	0	Y
315	2	50	0	0	0	Y
316	2	72	0	0	0	Y
317	2	85	0	0	0	Y
318	2	60	0	1	0	Y
319	2	61	0	0	0	Y
320	2	66	0	1	1	Y
321	2	69	0	1	1	Y
322	1	75	0	0	0	Y
323	1	54	0	1	0	Y
324	2	78	0	0	0	Y
325	2	53	0	0	0	N
326	1	64	0	0	0	N
327	2	62	0	1	0	Y
328	2	65	0	0	0	Y
329	1	69	0	1	0	Y
330	1	71	0	0	0	Y
331	2	68	0	1	0	Y
332	1	75	0	0	0	Y
333	1	50	0	0	0	Y
334	1	77	0	0	0	N
335	1	55	0	1	0	Y
336	1	64	0	1	0	Y
337	1	69	0	1	0	Y
338	1	68	0	1	1	Y
339	1	86	0	1	1	Y
340	2	78	0	0	0	Y
341	2	62	0	0	0	Y
342	1	81	0	1	0	Y
343	1	71	0	0	0	Y
344	1	79	0	0	0	N
345	1	82	0	0	0	Y
346	2	67	0	1	1	Y
347	2	63	0	1	0	Y
348	1	48	0	0	0	Y
349	1	67	0	1	0	Y
350	1	67	0	1	0	Y
351	1	64	0	0	0	Y
352	1	76	0	0	0	N
353	2	78	0	1	0	Y

354	1	75	0	1	0	Y
355	2	70	0	0	0	Y
356	1	64	0	1	0	N
357	1	79	0	0	0	Y
358	1	72	0	0	0	Y
359	2	63	0	0	0	Y
360	2	52	0	1	0	N
361	2	71	0	1	0	Y
362	1	81	1	0	0	Y
363	2	90	0	0	0	Y
364	2	56	0	1	0	Y
365	2	53	0	0	0	N
366	2	78	0	0	0	Y
367	1	72	1	1	1	N
368	1	76	1	0	0	N

居住地到医院距离<5KM 否=0 (大於 5KM) 是=1 (小於 5KM) distance_less_5km	眼别 which_eye	诊断 CNV=0 PCV=1 diagnosis	病程/M disease_history_month	光動力 無=0 有=1 PDT	药物类别 C=1 L=2 treatment_group
1	os	0	1	0	1
1	od	0	1	0	2
1	od	0	36	0	2
1	os	0	3	0	2
0	od	0	0.25	0	2
1	os	0	16	0	2
0	os	0	3	0	2
0	os	1	48	0	1
1	os	0	6	0	1
0	od	0	7	0	2
1	od	1	3	0	2
0	os	1	1	0	1
0	od	0	1	0	2
1	od	0	5	0	2
1	os	0	2	0	2
1	os	1	0.33	0	2
1	od	0	12	0	2
0	os	0	6	0	2
1	od	1	2	0	1
0	od	0	6	0	2
1	os	0	4	0	2
1	od	0	22	0	2
0	os	0	14	0	2
0	os	0	6	0	2
1	od	0	120	0	2
1	od	1	72	1	2
1	os	1	6	0	2
1	os	1	3	0	2
1	od	1	5	1	1
1	od	0	24	0	1
0	os	0	24	0	1
0	od	0	1	0	2
1	os	0	24	0	2
1	od	1	1	0	2
1	od	1	1	0	2
0	od	1	24	1	2
0	od	1	3	0	1
1	os	0	1	1	2
0	os	0	1	0	2
1	os	0	2	0	2
0	od	0	48	0	2
0	od	0	0.33	0	2
1	os	1	60	0	2
0	os	0	1	0	2
1	od	1	12	0	2
0	od	1	1	0	2
0	os	0	2	0	1

1	os	0	1	0	2
0	os	1	1	0	2
0	od	0	2	0	2
0	os	1	0.33	0	2
0	os	1	3	0	2
1	os	1	12	0	2
0	od	0	1	0	2
1	od	1	72	1	2
1	od	0	2	0	2
0	os	0	6	0	1
0	od	0	1	0	2
0	os	0	2	0	2
0	os	0	7	0	2
1	os	1	1	0	2
0	os	0	2	0	1
0	od	0	6	0	1
0	od	1	1	0	2
0	os	0	1	0	2
0	os	1	1	0	1
0	os	1	0.7	0	1
1	os	1	1	0	2
1	od	1	4	0	2
0	os	1	9	1	2
1	od	1	3	0	2
0	os	0	7	0	2
1	od	0	2	0	2
1	os	0	60	0	2
1	os	0	2	0	2
1	od	0	5	0	2
0	os	1	12	0	2
0	os	0	2	1	2
0	os	0	1	0	2
1	os	0	4	0	1
0	os	1	2	1	2
1	od	1	12	1	2
0	od	1	12	0	2
1	od	0	1	0	1
1	os	0	1	0	1
0	os	1	1	0	1
1	od	0	24	0	1
0	os	0	3	0	2
0	os	0	12	0	2
0	od	0	2	0	2
1	od	0	11	0	1
0	os	0	6	0	1
1	od	1	12	0	2
1	od	0	36	0	1
0	os	1	24	0	1
1	od	0	12	0	2
1	od	0	3	0	2
0	od	0	2	0	2

1	os	0	18	0	2
0	os	1	120	0	1
0	od	0	1	0	2
1	od	1	1	0	2
1	od	1	3	1	2
0	os	1	6	0	1
1	os	0	6	0	1
1	od	0	72	0	2
0	od	1	2	0	1
1	os	1	1	0	2
1	os	0	6	0	2
0	od	1	6	1	2
1	os	0	2	0	1
1	os	1	12	0	2
0	od	0	123	0	2
0	os	0	24	1	2
1	od	0	2	0	2
1	od	0	1	0	2
0	os	0	3	0	2
0	od	0	12	0	1
1	od	0	0.5	0	2
0	os	0	6	0	2
1	od	0	1	0	1
1	od	1	1	0	1
0	os	1	2	0	1
1	os	0	2	0	2
0	od	1	1	0	2
0	od	0	12	0	2
0	os	0	8	0	2
0	os	0	120	0	2
1	od	0	6	0	2
1	od	0	2	0	2
0	os	0	12	0	2
1	od	0	5	0	2
1	od	0	1	1	2
1	os	0	1	1	2
1	od	0	1	0	2
1	od	1	24	0	2
1	od	0	24	0	1
1	od	0	36	0	2
0	os	1	8	1	2
0	od	1	12	0	2
1	od	0	1	0	2
0	od	0	1	0	1
0	os	1	2	1	1
0	od	0	1	1	2
0	os	0	24	0	2
0	od	0	3	0	2
1	od	0	1	0	2
0	os	1	5	1	2
0	od	1	6	1	2

0	od	1	10	1	2
1	os	0	24	0	1
0	od	1	1	0	1
1	os	0	5	0	2
1	os	1	1	0	1
0	od	1	3	0	1
0	od	0	12	0	2
1	os	0	2	0	2
0	od	1	48	0	2
1	os	0	3	0	2
0	os	0	1	0	2
0	od	1	6	0	2
0	os	1	3	1	2
1	od	1	1	0	2
0	os	0	3	0	2
0	os	1	0.5	0	2
1	od	0	2	0	2
0	os	1	3	0	2
1	od	0	2	0	1
1	os	0	2	1	2
1	od	0	72	0	2
0	od	0	10	0	2
1	od	0	1	0	2
1	od	0	24	0	2
0	od	1	24	0	1
0	os	1	0.1	1	1
0	od	0	5	0	2
0	od	1	6	0	1
1	od	1	2	0	2
0	os	0	2	0	2
0	os	0	72	0	2
0	os	1	2	0	2
1	os	0	6	0	1
0	od	1	12	0	2
0	od	1	5	1	2
1	od	0	2	0	2
1	od	0	4	0	2
1	od	0	3	0	2
0	od	1	2	0	1
0	os	0	48	0	2
1	os	1	2	0	2
0	od	1	24	0	1
1	os	1	0.25	0	2
0	od	0	1	0	2
0	od	0	1	0	1
0	os	1	12	0	2
0	od	0	3	0	2
1	os	1	0.1	1	2
1	os	0	4	0	2
1	od	0	6	0	2
0	od	0	2	0	2
1	od	0	2	0	2

0	od	1	1	0	2
0	os	0	1	0	1
0	od	1	2	1	1
0	od	0	3	0	1
0	od	0	1	0	2
1	od	1	2	0	1
1	os	0	1	0	2
1	od	0	2	0	2
1	od	0	1	0	2
0	od	0	1	0	2
0	od	0	2	0	1
1	od	1	2	0	1
1	os	0	1	0	1
0	os	0	36	0	2
1	os	0	2	0	2
0	od	1	120	0	1
0	od	0	12	0	2
1	od	0	4	0	1
0	os	0	0.25	0	2
0	od	0	1	0	2
0	od	1	0.5	0	1
0	od	0	8	0	2
1	od	1	12	1	2
0	os	0	24	0	2
1	os	0	2	0	2
0	os	0	5	0	2
1	od	0	12	0	2
1	od	0	5	0	2
0	od	1	3	0	1
1	od	1	24	0	2
1	od	1	12	0	2
1	od	1	0.1	0	2
0	od	0	2	0	2
1	os	0	2	0	2
1	od	0	6	1	2
0	od	1	3	0	1
0	os	0	3	0	2
0	od	1	6	0	2
1	od	0	1	0	2
0	od	0	4	0	1
1	od	0	48	0	2
0	od	0	6	0	1
1	os	1	2	0	2
0	os	1	12	0	1
0	od	1	12	1	2
0	os	0	6	0	1
0	od	0	7	0	2
0	os	1	24	1	2
0	os	1	36	0	2
0	od	0	2	0	2

1	os	1	1	0	2
1	od	1	7	1	2
0	od	1	2	0	2
1	os	0	3	0	2
0	od	1	2	1	2
0	od	1	72	0	2
1	os	0	4	0	2
0	od	0	12	0	2
0	os	0	1	0	2
0	od	1	2	1	1
0	od	0	6	0	2
1	od	0	2	0	2
0	os	1	48	0	2
1	od	1	24	0	2
0	od	0	1	0	2
1	od	0	12	0	2
0	od	0	12	0	2
1	os	0	4	0	2
0	os	1	2	0	1
0	od	0	6	0	2
0	os	0	3	0	2
0	od	0	24	0	1
1	od	0	2	0	2
1	od	0	24	0	2
0	od	0	1	0	2
1	os	0	1	0	2
0	od	1	12	0	2
0	od	0	36	0	1
0	os	1	9	1	2
1	os	0	3	0	2
0	od	0	3	0	1
1	os	1	1	0	2
0	os	0	12	0	2
0	os	1	3	0	2
0	os	0	3	1	2
1	os	0	1	0	2
1	od	0	3	0	1
1	os	0	3	0	2
0	os	0	4	0	2
0	od	1	1	0	2
1	od	0	1	1	2
1	od	0	24	0	2
0	os	1	0.25	0	1
0	od	1	6	0	2
1	od	1	2	0	2
0	od	0	2	0	2
0	od	0	1	0	2
0	od	0	3	0	1
0	od	1	24	0	2
0	od	0	36	0	2
0	od	0	1	0	2
0	os	0	1	0	2

0	os	0	1	1	2
0	os	1	1	0	1
0	os	0	3	0	1
0	os	0	12	0	1
1	os	1	12	0	2
0	od	1	12	0	2
1	od	1	9	0	2
1	od	0	6	0	2
0	os	0	1	0	1
1	od	0	0.1	0	2
1	os	0	1	0	2
0	os	0	3	0	2
0	od	0	4	0	2
1	os	0	0.5	0	2
0	os	0	1	1	2
0	od	0	3	0	2
0	os	0	2	0	1
0	od	0	3	0	2
1	os	1	0.33	0	2
0	od	0	1	0	2
1	os	1	0.6	0	1
0	os	0	1	0	2
0	od	0	12	0	2
0	os	1	6	1	2
0	od	0	36	0	2
1	od	0	2	0	2
0	od	0	24	0	2
1	os	1	12	0	1
0	os	1	24	1	2
0	os	0	4	0	2
0	od	0	3	0	1
1	od	0	1	0	2
1	os	1	6	0	2
0	os	1	1	0	1
0	os	0	1	0	2
1	os	0	20	0	2
0	od	1	6	0	2
1	od	0	24	0	1
0	os	1	1	1	2
1	os	0	12	0	2
0	od	0	10	0	2
1	od	0	6	0	2
0	os	0	24	0	2
1	os	1	2	0	1
0	os	1	1	1	2
0	od	1	0.1	1	2
0	os	1	3	1	2
1	os	0	3	0	2
0	os	0	3	0	2
0	od	0	24	0	1

0	od	0	1	0	2
0	os	1	24	0	2
0	od	0	1	1	2
1	od	1	2	0	1
0	os	1	12	0	1
0	os	0	1	0	2
1	od	1	3	1	2
1	od	0	0.7	0	1
0	os	0	24	0	2
1	od	1	36	0	1
1	od	1	12	0	2
0	od	0	1	0	2
0	os	0	2	0	2
0	od	0	12	0	2
0	os	0	24	0	2

注射次数<br>No_of_injection	病灶大小/um<br>(空白 67 人)<br>area	基线VA<br>baseline_va	1mVA<br>one_m_va	12mVA<br>one_year_va	第1個月 VA 變化<br>one_m_va_change	12個月 VA 變化<br>one_year_va_change
1	1453	48	60	62	12	14
1	3043	23	40	48	17	25
2	3362	45	42	40	-3	-5
3	1087	48	48	57	0	9
1	2236	20	22	23	2	3
3	1444	40	40	28	0	-12
4	1482	40	40	55	0	15
1	1831	45	48	48	3	3
6	2783	55	60	60	5	5
5	1778	25	25	25	0	0
3	2347	22	22	25	0	3
1	3876	40	60	50	20	10
1	3420	21	24	24	3	3
4	1991	55	68	68	13	13
3	799	62	62	62	0	0
1	484	25	62	62	37	37
1	2145	25	48	34	23	9
3	1963	21	25	25	4	4
2	2085	55	48	55	-7	0
4	2121	25	75	40	50	15
3	4549	40	40	47	0	7
8	1435	68	68	62	0	-6
3	1305	25	24	28	-1	3
1	1926	23	20	23	-3	0
1	1745	20	23	20	3	0
2	883	22	23	23	1	1
3	2261	23	24	48	1	25
3	2068	23	24	48	1	25
3	2057	45	40	50	-5	5
1	1864	22	24	24	2	2
1	1383	20	20	20	0	0
1	623	55	70	62	15	7
1	2492	25	25	25	0	0
1	2068	23	25	40	2	17
1	2663	24	75	28	51	4
1	3712	22	22	22	0	0
1	2077	20	20	20	0	0
1	4592	23	23	23	0	0
2	2981	20	20	20	0	0
3	2373	20	20	24	0	4
1	827	40	40	40	0	0
1	2034	20	20	20	0	0
1	4953	25	25	25	0	0
1	1756	40	48	45	8	5
1	1434	20	28	48	8	28
1	677	25	40	30	15	5
2	532	25	40	48	15	23

2	1754	22	22	24	0	2
2	692	75	60	64	-15	-11
2	3400	23	23	23	0	0
1	2833	40	40	40	0	0
3	2423	24	24	48	0	24
4	2765	55	55	55	0	0
1	666	72	72	70	0	-2
2	4625	22	20	22	-2	0
3	2324	48	40	48	-8	0
2	2466	20	20	20	0	0
1	1007	40	48	55	8	15
2	1369	25	40	57	15	32
3	3722	20	40	25	20	5
2	1321	40	48	60	8	20
2	629	48	48	55	0	7
2	2946	20	24	24	4	4
2	4252	22	25	45	3	23
2	3334	40	48	60	8	20
2	92	25	48	48	23	23
1	3426	22	40	36	18	14
2	3066	40	40	60	0	20
3	2442	25	20	25	-5	0
8	1289	55	55	55	0	0
3	3234	23	23	48	0	25
5	2267	48	60	55	12	7
3	3201	62	55	62	-7	0
3	2360	23	23	25	0	2
3	2484	20	22	23	2	3
4	2468	28	48	60	20	32
3	3246	25	40	48	15	23
2	2382	35	48	60	13	25
1	4990	20	20	20	0	0
4	847	24	25	40	1	16
3	657	75	48	56	-27	-19
2	404	40	55	55	15	15
3	3356	25	25	25	0	0
1	2364	40	40	40	0	0
1	657	55	55	55	0	0
1	2866	48	48	48	0	0
1	2663	48	25	25	-23	-23
3	1375	25	24	40	-1	15
4	3322	40	40	47	0	7
2	1954	40	62	62	22	22
7	1473	22	22	45	0	23
5	1145	25	40	48	15	23
2	2788	25	40	36	15	11
1	1802	25	25	25	0	0
1	4756	25	25	25	0	0
3	3794	23	25	28	2	5
3	2853	24	25	25	1	1
3	5478	20	20	23	0	3

3	392	40	72	70	32	30
1	2785	23	24	25	1	2
1	957	25	25	28	0	3
1	3982	24	40	42	16	18
1	6223	24	23	20	-1	-4
1	3469	20	20	20	0	0
3	5410	20	20	20	0	0
3	6666	20	25	25	5	5
3	3927	23	23	24	0	1
1	4429	23	28	40	5	17
1	1887	20	48	39	28	19
1	1093	25	25	25	0	0
3	984	25	25	44	0	19
1	1113	28	40	36	12	8
3	3007	20	40	31	20	11
1	6093	22	22	22	0	0
3	4572	23	23	47	0	24
1	1539	28	45	40	17	12
3	3131	40	48	60	8	20
2	4381	25	40	38	15	13
1	4270	22	24	25	2	3
1	3500	22	62	47	40	25
1	2109	25	55	46	30	21
1	3154	55	62	60	7	5
2	4699	20	24	24	4	4
3	3741	24	25	48	1	24
1	897	48	70	63	22	15
3	2319	40	45	45	5	5
3	3201	55	48	48	-7	-7
1	6259	25	75	70	50	45
3	4427	21	21	24	0	3
3	3962	23	23	28	0	5
4	9007	22	25	25	3	3
1	1446	20	20	20	0	0
2	4717	25	40	38	15	13
2	1442	23	40	48	17	25
2	1650	35	48	48	13	13
4	1200	20	20	22	0	2
1	1431	25	40	48	15	23
4	2570	23	23	25	0	2
3	1382	40	62	64	22	24
3	6231	25	25	23	0	-2
1	2634	40	48	48	8	8
1	2282	23	28	28	5	5
1	3087	25	48	56	23	31
1	3850	23	25	25	2	2
1	370	25	40	40	15	15
3	3442	23	22	25	-1	2
1	2570	55	55	40	0	-15
3	532	25	25	40	0	15
2	2644	25	28	25	3	0

6	539	48	48	45	0	-3
3	2506	40	40	40	0	0
1	1487	25	40	40	15	15
5	416	24	23	32	-1	8
1	2284	20	20	20	0	0
2	1468	21	30	35	9	14
3	1380	20	20	22	0	2
3	107	35	48	48	13	13
3	2073	23	25	23	2	0
2	2833	23	40	45	17	22
1	920	25	40	40	15	15
1	679	25	25	25	0	0
3	2367	25	40	38	15	13
1	4247	21	22	23	1	2
3	3923	45	48	60	3	15
1	416	20	40	35	20	15
3	623	75	40	48	-35	-27
2	3768	24	40	37	16	13
3	454	24	25	40	1	16
2	1830	20	25	25	5	5
3	3389	22	24	24	2	2
1	4263	23	24	24	1	1
1	1478	48	62	55	14	7
2	3043	20	25	24	5	4
2	1125	40	25	23	-15	-17
1	107	24	48	48	24	24
3	4245	25	25	25	0	0
3	609	20	25	25	5	5
2	2522	24	24	45	0	21
2	1963	22	25	45	3	23
1	1762	22	22	22	0	0
1	3456	25	25	25	0	0
2	2874	24	40	23	16	-1
3	5293	24	40	48	16	24
4	2897	23	25	35	2	12
2	1953	55	55	68	0	13
1	2282	25	24	25	-1	0
2	3126	20	25	40	5	20
1	4415	25	20	25	-5	0
1	2935	45	55	50	10	5
2	3286	23	24	40	1	17
1	3958	40	55	55	15	15
1	2347	40	48	44	8	4
1	3388	25	25	28	0	3
1	3247	40	60	45	20	5
5	2774	55	60	68	5	13
3	892	72	70	72	-2	0
1	421	20	20	20	0	0
4	1893	40	25	35	-15	-5
3	2082	23	25	55	2	32
3	1615	24	25	40	1	16
3	679	55	40	44	-15	-11

1	3766	24	24	24	0	0
1	2482	21	25	25	4	4
1	1612	22	22	22	0	0
3	992	24	25	48	1	24
1	1091	40	40	48	0	8
1	6465	20	20	20	0	0
1	2874	25	40	40	15	15
1	1357	25	25	29	0	4
1	977	22	24	24	2	2
2	782	48	48	55	0	7
2	1757	25	25	48	0	23
1	4964	24	29	29	5	5
1	2738	60	62	62	2	2
1	1722	45	55	52	10	7
2	2058	25	25	48	0	23
1	3310	25	25	40	0	15
2	2115	25	25	38	0	13
3	4545	20	20	20	0	0
1	1236	60	62	62	2	2
2	2891	21	70	50	49	29
1	1406	55	73	60	18	5
6	657	62	62	62	0	0
3	2866	23	24	50	1	27
2	4490	22	25	25	3	3
3	1438	24	25	48	1	24
4	2171	48	62	60	14	12
1	1542	22	24	25	2	3
3	1375	55	48	58	-7	3
1	1821	55	48	55	-7	0
4	1333	45	48	38	3	-7
3	7597	20	20	22	0	2
1	2061	22	25	23	3	1
2	2622	21	25	24	4	3
3	782	40	40	48	0	8
4	1446	25	48	55	23	30
2	1305	24	25	25	1	1
1	2188	24	24	24	0	0
1	5530	30	38	35	8	5
1	1851	22	23	25	1	3
1	2419	40	40	48	0	8
1	2445	21	25	24	4	3
3	784	25	48	50	23	25
1	4087	24	29	29	5	5
1	518	24	22	28	-2	4
2	4637	25	24	23	-1	-2
1	2007	62	62	66	0	4
3	2970	22	23	22	1	0
2	3043	35	48	65	13	30
2	1370	40	40	49	0	9
3	1656	40	40	55	0	15

2	3766	25	40	48	15	23
2	5134	22	22	40	0	18
2	936	21	24	24	3	3
3	1342	28	48	55	20	27
4	4643	25	25	25	0	0
1	1900	28	36	32	8	4
5	1125	25	25	48	0	23
3	1672	35	48	43	13	8
1	2709	40	60	60	20	20
3	4631	22	25	24	3	2
2	1140	25	48	60	23	35
3	1380	40	55	48	15	8
1	3066	20	20	20	0	0
2	2200	40	55	45	15	5
1	515	25	28	32	3	7
2	2385	25	33	40	8	15
3	2018	40	48	45	8	5
3	669	62	70	68	8	6
1	1891	55	55	55	0	0
3	1615	22	22	40	0	18
3	883	25	40	68	15	43
1	1901	25	40	38	15	13
3	1463	24	25	25	1	1
1	1846	23	40	35	17	12
1	1641	68	68	70	0	2
3	638	40	68	55	28	15
2	7474	22	23	26	1	4
1	6523	20	21	21	1	1
2	2702	23	25	30	2	7
3	4490	25	24	40	-1	15
3	1428	23	25	55	2	32
2	6285	40	40	46	0	6
1	2847	25	25	25	0	0
1	2370	35	40	45	5	10
3	2110	24	24	40	0	16
1	2857	25	48	45	23	20
3	1900	25	25	45	0	20
3	1503	25	40	48	15	23
4	1329	40	55	48	15	8
1	3073	25	25	40	0	15
1	1643	25	40	40	15	15
2	2374	20	22	22	2	2
1	1093	23	28	28	5	5
4	723	28	20	28	-8	0
2	1896	40	40	48	0	8
2	3452	21	22	30	1	9
2	893	55	48	60	-7	5
3	905	21	23	48	2	27
1	1494	25	40	40	15	15
1	2368	23	25	57	2	34
1	2382	24	25	35	1	11
1	1722	25	25	27	0	2

1	2093	60	48	60	-12	0
2	1471	48	40	44	-8	-4
3	2183	25	40	25	15	0
1	1867	20	22	22	2	2
1	1491	48	60	40	12	-8
1	2364	62	60	60	-2	-2
4	946	24	28	48	4	24
2	1600	21	23	24	2	3
1	2370	25	25	40	0	15
1	3548	40	48	45	8	5
1	2959	20	20	20	0	0
3	285	20	45	70	25	50
3	2977	23	23	23	0	0
1	3861	24	24	24	0	0
1	1059	22	25	25	3	3
3	4253	23	24	55	1	32
1	4503	62	70	66	8	4
3	3371	20	20	25	0	5
1	3208	25	40	32	15	7
1	1487	25	25	45	0	20
1	1844	70	65	68	-5	-2
1	4492	24	28	45	4	21
3	1140	55	48	48	-7	-7
4	4566	21	22	22	1	1
2	4998	25	25	35	0	10
3	962	40	40	48	0	8
3	4547	48	48	48	0	0
2	3005	46	48	53	2	7
4	1975	21	24	22	3	1
3	2902	25	25	21	0	-4
1	3036	25	25	25	0	0
1	357	70	70	72	0	2
1	2195	55	60	60	5	5
1	953	55	60	57	5	2
3	1641	23	40	25	17	2
4	2249	25	40	40	15	15
1	3463	20	23	22	3	2
1	1435	32	35	35	3	3
1	3548	22	40	40	18	18
3	1148	48	55	52	7	4
2	3795	24	24	24	0	0
1	12700	28	35	35	7	7
2	1343	20	24	23	4	3
2	1205	25	25	25	0	0
1	3876	25	25	40	0	15
3	1853	25	40	25	15	0
3	3765	33	48	60	15	27
4	887	68	62	70	-6	2
1	4845	25	25	25	0	0
3	3184	23	24	24	1	1

2	3342	25	24	40	-1	15
2	1444	23	25	35	2	12
1	1343	62	62	62	0	0
2	2043	45	48	40	3	-5
4	3194	23	25	25	2	2
1	1831	48	55	55	7	7
3	2513	25	42	45	17	20
1	723	25	35	35	10	10
2	3059	20	40	40	20	20
2	6549	20	22	22	2	2
4	1667	40	20	22	-20	-18
1	3000	21	21	21	0	0
2	2208	40	48	55	8	15
2	3297	20	40	40	20	20
2	6493	22	22	22	0	0

基线OCT<br>baseline_oct	1mOCT<br>one_m_oct	2mOCT<br>two_m_oct	3mOCT<br>three_m_oct	6mOCT<br>six_m_oct
244	197		187	
348	268	225	199	187
806	218	206		
313	280	288	238	213
873	672	692		476
225	250	206	121	311
285	249	254	214	201
237	139	164		161
255	198	188	168	149
301	333	253	244	290
596	435	359	215	178
753	421			345
565	466		387	323
275	222	282	275	242
238	256	290	247	225
446	187			
232	187		213	
959	371	312	315	
794	611		576	321
266	237	219	204	
425	433	374	376	
253	301	319	235	197
954	757	548	359	254
353	345		263	
690	765		575	
305	278	294		
254	236	166	186	
387	311	167	178	183
415	356	336	530	
826	647		324	228
213	213		253	
460	364	501		314
349	322		267	287
176	169	175	172	
531	379	268	226	288
575	468		437	
537	537		256	
100	119	118		110
493	323	265	547	
388	388	330	287	244
314	314			245
216	193	194	162	176
496	464			364
214	188	204		175
735	272		168	
355	354	265		
246	225	276		226

312	275	257	234	209
436	245	231		
314	276	325	316	350
607	589		234	
371	328..77	274	165	140
248	169	167	172	180
138	146		133	147
1091	291	224		
257	250	253	198	210
283	216	197		
384	284	242	288	208
216	193	194	162	176
542	498	553		329
257	246	205	184	
287	217	195		
491	375	234	258	324
401	299	289	277	223
298	240	202	211	199
323	205	177		
422	359	341		273
295	343	268	230	211
454	446	282	328	
703	600	813	722	430
1074	930	800	420	246
198	221	192		187
227	230	211	188	184
468	477	547	394	
634	636	457	387	266
425	354	341		290
406	443		324	267
1001	256	230	194	
646	426		680	589
578	422	446	607	470
599	299	197	191	
500	426			272
310	258	149	145	
290	240	246	223	
143	155	148	134	156
273	245	233	219	
308	222		231	
766	678		322	228
343	380		334	253
456	392	225	196	
199	222	193	193	188
808	475			422
616	473		278	
864	789			
565	555	442		392
339	182	161		197
343	237	222	210	205
201	188	195		165

366	218	286		
372	283			267
254	225	192		177
351	256	288	279	226
343	425		326	
567	622			
522	480	368	285	
949	516	259	216	
276	182	197		181
354	244	229		
295	268	229		
350	228			
510	458	417	319	246
728	150			
273	245	233	219	
313	339			231
380	343	332	247	225
251	214		224	238
377	289	226	198	152
430	375			538
525	364		375	
944	802			947
406	308			
352	424	442		564
395	336			
166	174	187	179	184
308	222		231	
418	242	242	287	221
416	329	256	280	261
648	489			
357	377	279	298	258
276	247	230	191	207
748	488			
418	225	233	186	221
216	198	188	199	177
269	208	224	188	198
306	264	318	369	
1632	1632	655	288	240
358	267			312
936	875	721	600	505
243	190	161	207	167
418	336			446
250	203	178		206
891	672		487	
418	225	233	186	221
203	214	224		186
195	195		194	187
289	235	182	178	164
224	197	209		
475	423	444	512	476
664	323	346	219	123

327	319	318	229	329
246	240		242	
475	364			
295	256	293	237	215
672	746			532
292	144			239
1221	386	259		
486	222	235	174	187
605	512	314	295	
594	291	257	210	228
376	272			
416	390	408		
416	288	218	390	294
123	138	113	97	
370	254	211	184	167
590	478		324	
277	222	192	188	178
384	170	173		
365	348	383	307	286
850	859	623		592
272	226	194		177
520	300	266	197	178
406	245	298	209	193
582	618	645		324
165	160	226	299	160
415	169	156	178	
678	485			282
1098	703	397	345	381
433	388	253	224	232
392	301	266	197	178
316	309		242	
479	367	244		342
471	388			452
395	214	224	221	346
135	115	108		223
245	298	209	193	
199	186			
296	372	289	234	221
637	664	348		452
186	275	249		184
766	620	263		244
337	681			
233	217		182	200
860	476		386	
353	371		287	
353	332	360	210	243
237	184	183	167	154
726	445	148		
529	735	235	451	225
597	527	559	619	518
243	254	211	187	175
277	351	258	217	202

336	352	268	221	201
557	264		274	
290	120			
673	644	455	258	187
266	223		222	
574	377	264		
385	254	276	273	
853	779		474	
444	381			275
224	244	202	198	224
279	541	279	233	
479	425		544	
267	202	223		211
384	310			
483	398	335	284	259
424	246			
375	304	315	256	252
766	370	226	350	318
248	214			
375	222	321		
360	435	278	266	
198	185	177	180	169
382	188	176		221
453	216		280	
327	304	312	251	238
349	229	194	251	178
623	467			
235	284	194		
688	641	589		
347	215		283	523
1306	1245	688		149
772	148	116		
765	649	555	721	242
486	387	211	199	180
308	196	154	208	154
1238	676		433	536
449	428			
200	212			180
540	501			
334	321	332	265	272
613	364			562
175	172		172	
245	180	213	176	190
434	447			
699	203	199		
1059	965		868	
720	496	506	544	
355	460	211		
416	219	245		
359	322	333	296	268

335	282	244	201	186
375	322	302	276	225
433	208			218
307	201	213	187	138
365	264	211	233	212
428	426		257	
387	350	328	254	224
304	265	325	243	197
239	177	198		174
335	264			330
257	223	202	222	199
304	259	222	188	198
571	490		353	
252	214	219	218	211
531	498		333	376
254	235	202		197
604	218	212		213
330	239	235	434	218
593	477			487
305	233	235	224	
409	225	156	149	156
458	474		353	
517	312	298	257	238
353	264			
293	232		197	
264	166	298	232	230
645	281	371		
208	257		242	
170	165		224	323
554	462	367	183	172
365	287	219	158	166
502	354	267	246	
406	363		242	
467	165		180	185
521	492	316	279	189
398	257	202		235
242	266	248	192	168
767	272	235	176	153
367	322	277	298	
801	453	264		232
275	235	258		221
365	286	208	196	263
494	322	277	298	
513	421	285	156	152
321	215	401	445	706
288	253	223	205	
235	227	214	187	192
182	130	123	110	
214	209	167	153	
633	296		270	
214	223		207	
206	218		235	

330	259	267		226
226	175	225		
339	315		444	
648	449	383		
354	275			
223	294			
768	614	510	443	402
539	238			419
275	236		214	
831	861		349	
973	783		1360	
804	131	133	135	155
257	215	408	347	
717	654	313	253	422
489	329	253		
599	361	357	248	173
275	232	245	174	184
325	304	287	220	203
1218	744		976	
648	449	383		
334	472		353	
422	289	252		
363	273	261	317	254
601	442	276	215	461
375	355	275		264
573	436	301	212	250
370	190			
204	164		233	250
254	288	250		
313	162	423	334	222
148	264	156		134
201	184	156		147
267	196	273	254	183
685	418		575	
457	247	207		
272	246	267	261	234
286	252			
254	245	267	225	243
374	223			203
290	225		235	242
338	363	290		253
193	163	154		
679	443	502		363
256	163	154		198
684	339	289		227
305	670		391	
456	406	567	287	232
198	187	193	246	190
478	329	256	366	
792	663		820	

287	309	267	223	257
264	203	188	190	188
303	249	227		249
527	527	447		
1088	574	195	180	
332	270	257		
480	223	192	163	182
205	219			
297	238	253		
372	518		448	
290	654		481	434
970	872		405	
295	285	236	217	198
676	618			
415	290	254		

12mOCT<br>one_year_oct	第1個月 OCT 變化<br>one_m_OCT_change	12個月 OCT 變化<br>one_year_OCT_change
192	-46	-51
203	-80	-145
242	-588	-564
224	-33	-89
476	-201	-397
187	25	-38
185	-36	-100
309	-98	72
166	-56	-89
198	32	-103
195	-161	-401
345	-332	-408
323	-99	-242
225	-53	-49
219	18	-19
217	-259	-229
213	-45	-19
315	-588	-644
321	-183	-473
184	-29	-82
786	8	361
216	48	-38
316	-197	-638
263	-8	-90
675	75	-15
282	-27	-23
184	-19	-71
172	-76	-216
353	-59	-62
364	-179	-462
224	0	11
314	-96	-146
287	-27	-62
172	-7	-4
237	-152	-294
437	-107	-138
256	0	-281
110	19	10
547	-170	54
232	0	-156
245	0	-69
176	-24	-40
364	-32	-132
165	-26	-48
256	-463	-479
277	-1	-78
189	-22	-57

133	-37	-179
342	-191	-94
350	-38	36
387	-18	-220
153	#VALUE!	-218
161	-79	-87
174	8	37
387	-800	-704
193	-7	-64
216	-67	-67
179	-99	-204
176	-24	-40
329	-44	-213
177	-12	-81
219	-70	-68
368	-116	-123
199	-103	-203
176	-59	-122
189	-118	-134
249	-63	-173
179	48	-117
328	-8	-126
418	-103	-285
176	-144	-898
201	23	3
168	3	-59
318	9	-150
307	3	-327
247	-71	-178
267	37	-139
154	-744	-847
472	-220	-174
358	-156	-220
191	-300	-408
272	-74	-228
145	-52	-165
209	-51	-82
145	12	2
243	-28	-30
232	-86	-76
155	-87	-611
253	37	-90
204	-64	-252
202	23	3
516	-333	-292
293	-143	-323
359	-75	-505
392	-10	-173
186	-157	-153
227	-106	-115
179	-13	-21

198	-148	-168
257	-89	-115
197	-29	-56
226	-94	-125
273	82	-70
329	55	-238
285	-42	-237
216	-433	-733
181	-94	-95
193	-110	-161
282	-27	-13
293	-122	-57
245	-52	-265
283	-578	-445
243	-28	-30
231	26	-82
199	-37	-181
324	-36	73
135	-88	-242
242	-55	-188
392	-161	-133
916	-142	-28
327	-98	-79
590	72	238
248	-59	-147
166	8	0
232	-86	-76
314	-176	-104
488	-87	72
329	-159	-319
240	20	-118
181	-29	-96
646	-260	-102
323	-193	-95
165	-18	-51
188	-61	-81
318	-42	12
240	0	-1392
312	-91	-46
492	-61	-444
641	-53	398
372	-82	-46
197	-47	-54
387	-218	-503
323	-193	-95
157	11	-46
187	0	-8
126	-55	-163
188	-27	-37
441	-52	-34
162	-341	-502

273	-8	-54
248	-6	2
168	-111	-307
188	-39	-108
529	74	-143
189	-148	-103
233	-835	-988
186	-264	-300
970	-93	365
197	-304	-397
241	-104	-135
332	-26	-84
287	-128	-129
123	15	0
153	-115	-216
479	-112	-111
194	-55	-84
182	-214	-202
224	-16	-141
429	9	-421
197	-46	-75
178	-220	-342
234	-161	-172
324	36	-258
160	-5	-5
223	-246	-192
324	-193	-354
459	-395	-639
199	-45	-234
178	-91	-214
282	-7	-34
386	-112	-93
342	-83	-129
346	-181	-49
223	-20	88
163	53	-82
178	-13	-21
202	77	-94
452	27	-185
198	89	12
243	-146	-523
342	344	5
224	-16	-9
222	-384	-639
340	18	-13
196	-21	-157
163	-53	-75
286	-281	-440
194	206	-334
582	-70	-15
175	11	-68
188	74	-90

193	16	-143
374	-293	-183
153	-170	-137
2685	-28	2013
203	-43	-63
378	-197	-196
224	-131	-162
683	-74	-170
332	-63	-112
193	19	-32
165	262	-114
483	-54	4
211	-65	-56
310	-74	-74
214	-86	-270
266	-178	-158
295	-71	-80
318	-396	-448
214	-34	-34
301	-153	-74
341	75	-19
182	-13	-15
232	-194	-150
374	-237	-79
211	-24	-117
162	-120	-186
373	-156	-250
239	49	4
458	-47	-230
736	-132	389
254	-61	-1052
252	-624	-520
463	-116	-302
206	-98	-279
168	-113	-141
518	-562	-720
363	-21	-86
215	12	15
367	-39	-173
284	-13	-50
478	-249	-135
185	-3	10
225	-65	-20
364	13	-70
328	-496	-371
842	-94	-217
345	-224	-375
264	105	-91
267	-197	-149
237	-37	-122

193	-53	-142
202	-53	-173
213	-225	-220
152	-106	-154
176	-101	-189
352	-2	-76
277	-37	-110
236	-39	-68
166	-63	-73
276	-71	-59
181	-34	-76
173	-45	-130
255	-81	-316
211	-38	-41
353	-33	-178
214	-19	-40
216	-386	-388
228	-91	-102
487	-116	-106
217	-72	-88
167	-184	-242
356	16	-102
218	-206	-300
436	-89	83
167	-61	-126
244	-98	-21
436	-364	-209
324	49	116
216	-5	46
199	-92	-355
185	-78	-180
208	-148	-293
324	-43	-82
232	-302	-235
163	-28	-357
135	-141	-263
142	23	-100
160	-495	-606
258	-45	-109
232	-348	-569
183	-39	-91
243	-79	-122
258	-172	-236
134	-92	-379
674	-106	353
192	-34	-96
184	-8	-51
116	-52	-66
174	-5	-40
236	-337	-397
189	10	-25
194	12	-12

207	-71	-123
245	-51	19
353	-24	14
252	-199	-396
345	-79	-9
256	71	33
402	-154	-366
419	-301	-120
198	-38	-77
475	30	-356
982	-190	9
150	-673	-654
355	-42	98
546	-63	-171
237	-160	-251
132	-238	-467
215	-43	-60
233	-21	-92
854	-474	-364
252	-199	-396
364	138	30
187	-132	-234
275	-90	-88
183	-159	-418
246	-20	-129
235	-137	-338
247	-180	-123
218	-40	14
398	34	144
222	-151	-91
146	116	-2
153	-17	-48
224	-71	-43
464	-267	-221
969	-210	512
227	-27	-45
237	-34	-49
226	-9	-28
236	-150	-138
242	-65	-48
253	25	-85
164	-30	-29
363	-236	-316
154	-93	-102
226	-345	-458
214	365	-91
198	-50	-258
175	-11	-23
254	-149	-224
820	-129	28

206	21	-81
216	-61	-48
249	-54	-54
324	0	-203
180	-514	-908
203	-62	-129
153	-257	-328
248	14	43
228	-59	-69
257	146	-115
596	364	306
405	-98	-565
209	-9	-86
432	-58	-244
254	-125	-161
